# Early warning model and prevention of regional financial risk integrated into legal system

**DOI:** 10.1371/journal.pone.0286685

**Published:** 2023-06-02

**Authors:** Yanyu Zhuang, Hua Wei

**Affiliations:** 1 KoGuan School of Law, Shanghai Jiao Tong University, Shanghai, China; 2 Antai College of Economics and Management, Shanghai Jiao Tong University, Shanghai Development Strategy Research Institute, Shanghai, China; University of Malta, MALTA

## Abstract

In order to improve the laws and regulations of the financial system, in the construction of laws and regulations, the traditional financial risk Early Warning (EW) model is optimized. The financial prevention and control measures with legal protection are implemented to warn the financial risks, which plays an important role in the construction of the rule of law in the Financial Market (FM) and the establishment of financial risk prevention and control laws and regulations. This paper combines the deep learning model and the Markov regime Switching Vector Auto Regression (MS-VAR) model and constructs a regional financial risk EW model from the following aspects: macroeconomic operation EW indicators, regional economic risk EW indicators, regional financial institution risk EW indicators. The model is empirically researched and analyzed. The results show that the fluctuation trend of the macroeconomic pressure index in the time series is relatively large, and the overall fluctuation of the regional economic pressure index is small, and fluctuates around 0 in most periods. After the financial crisis, local governments stepped up their supervision of non-performing corporate and household loans. From 2011 to 2018, the non-performing loan ratio began to decline, and the overall fluctuation of the regional financial comprehensive stress index was small, fluctuating around 0. Due to the lack of legal regulation, from the perspective of the regional economy, the risk level is more likely to change from low risk to moderate risk, while the risk status is less likely to change from high risk to moderate risk. From the perspective of regional financial institutions, the probabilities of maintaining low risk and moderate risk are 0.98 and 0.97, respectively, which is stronger than maintaining the stability of high risk. From the perspective of the state transition of the regional financial risk composite index, the probability of maintaining low risk and high risk is 0.97 and 0.93, which is higher than maintaining the stability of medium risk. The Deep Learning (DL) regional financial risk EW MS-VAR model has strong risk prediction ability. The model can better analyze the conversion probability of regional financial risk EW index and has better risk EW ability. This paper enhances the role of legal systems in financial risk prevention and control. The regional financial risk EW model incorporating financial legal indicators can better describe the regional financial risk level, and the EW results are basically consistent with the actual situation. In order to effectively prevent financial risks and ensure the safety of the financial system, it is recommended that the government improve local debt management, improve financial regulations and systems, and improve the legislative level of financial legal supervision.

## Introduction

In the decades of social and economic development in China, the financial industry plays a vital role in the market economy and is the key to the market economy. In order to improve the laws and regulations of the financial system, the traditional financial risk early warning model is optimized in the construction of laws and regulations. The implementation of legally protected financial prevention and control measures and early warning of financial risks play an important role in the legal construction of Financial Market (FM) and the establishment of laws and regulations on financial risk prevention and control. FM malfunctions occur from time to time, and the risk probability increases significantly. Financial risk has the characteristics of high risk and high crisis, which is likely to cause serious harm to the market economy [1]. In particular, Regional Financial Risks (RFRs) have strong linkage and spread, and it is easy to induce national financial risks and even global financial crisis through ripple effect [2]. Under the downward pressure of huge Economic Growth (EG), various uncertain factors appear. Therefore, Early Warning (EW), prevention and control of RFRs have become an important task of macro-control in China [3]. Therefore, it is essential to emphasize the implementation of effective financial risk prevention measures. Meanwhile, relevant laws and regulations must be improved to make FM develop harmoniously and orderly.

Although national legal supervision can effectively monitor risks, effective financial risk EW measures can make the FM develop more harmoniously and orderly, make the whole market economy run more smoothly, and bring higher economic benefits to the society [4]. Strengthening RFRs-oriented EW has important practical significance for preventing financial risks and ensuring stable regional EG [5]. Financial behavior is highly related to the economy and society. All walks of life have paid more attention to the risks in the financial field. As a popular technology in Artificial Intelligence (AI), Deep Learning (DL) can model abstract high-level features of various data with multiple processing layers and nonlinear transformation [6]. Meanwhile, DL can find appropriate and effective features from complex data by processing big data, learning features through training, and using multi-layer perceptron models to supervise unsupervised data learning. The deeper the model is, the more accurate the feature expression will be [7]. Chen et al. (2020) used Deep Neural Network (DNN) to model the evacuation of subway station buildings, and carried out simulation experiments. Comparing the Convolutional Neural Network (CNN) model with the pre-training model by classifying data sets, the accuracy and training speed of the proposed model are verified [8]. Chen et al. (2021) used DL technology to model the network security system of smart cities, reducing the network security risks [9]. DL can also be used in financial analysis to predict commodity prices, financial events, financial risks and other hot issues. For example, Zhou et al. (2021) applied DL to the financial risk early warning of real estate enterprises. They took real estate as an example to make a concrete demonstration and analysis [10]. For RFRs-oriented electronic warfare, scholars have also conducted relevant research on risk causes, propagation paths, indicators, and model selection [11]. Du et al. (2021) established a scientific and effective oriented electronic warfare system based on Big Data Technology (BDT). They integrated a lot of data and introduced related risk index [3]. In selecting financial risk EW indexes, the Evaluation Index System (EIS) mainly focuses on currency crises, bond crises, or banking crises and fails to reflect the systemic financial risk fully. Moreover, the traditional financial risk EW system is mainly based on the linear model [12].

Based on the above theory, this paper improves the selection of financial risk EW indicators and risk EW models. Section 1 describes the purpose of writing the article and literature review. In Section 2, the characteristics, incentive mechanism and risk factors of regional finance are summarized, and the regional financial risk EW system is constructed by using DL model and Markov regime switching vector autoregression (MS-VAR) model. Then, the regional financial risk EW index and its comprehensive index are constructed, and the regional financial risk EW MS-VAR model based on DL is constructed. Section 3 analyzes the comprehensive index of economic stress from two aspects: macro-economy and regional economy, and regional financial stress from two aspects: regional financial institutions and regional finance. This model is used to test the EW of risks in different dimensions and predict regional financial risks. Section 4 summarizes the main results of this study and analyzes the future research direction. The innovation of this paper is to integrate DL model and financial legal system into the construction of regional financial risk EW model, and analyze the comprehensive indicators of regional financial risk EW model from many aspects. The design aims to make the EW of RFRs conform to the development characteristics of the local financial industry and improve the predictability of the EW model of financial risk. This discovery provides a reference for subsequent scholars to study financial risk EW.

## Theoretical research and method design of regional financial risk

### Overview of RFRs theory

#### 1. Basic concepts and characteristics of RFRs

RFRs is a variety of risks in the financial industry within a certain economic range. According to the impact region and performance characteristics, financial risks are divided into macro, regional, and micro levels. In particular, RFRs belongs to the meso level [13].

The formation of financial risks within a specific range generally has three-level factors. The micro-level risk spreads within a specific range and has the characteristics of top-to-bottom development. Risks spreading in some highly different economic ranges belong to horizontal risk. Finally, the macro-level risks accumulate and spread in the financial industry system [14]. RFRs have the general characteristics of financial risks, such as objective existence, controllability, and negative impact, and [15] at the same time, have unique characteristics. For example, the RFRs formation mechanisms are special. The risk harms limited areas, and there is a remarkable effect in RFRs-oriented EW [16].

**Fig 1:**
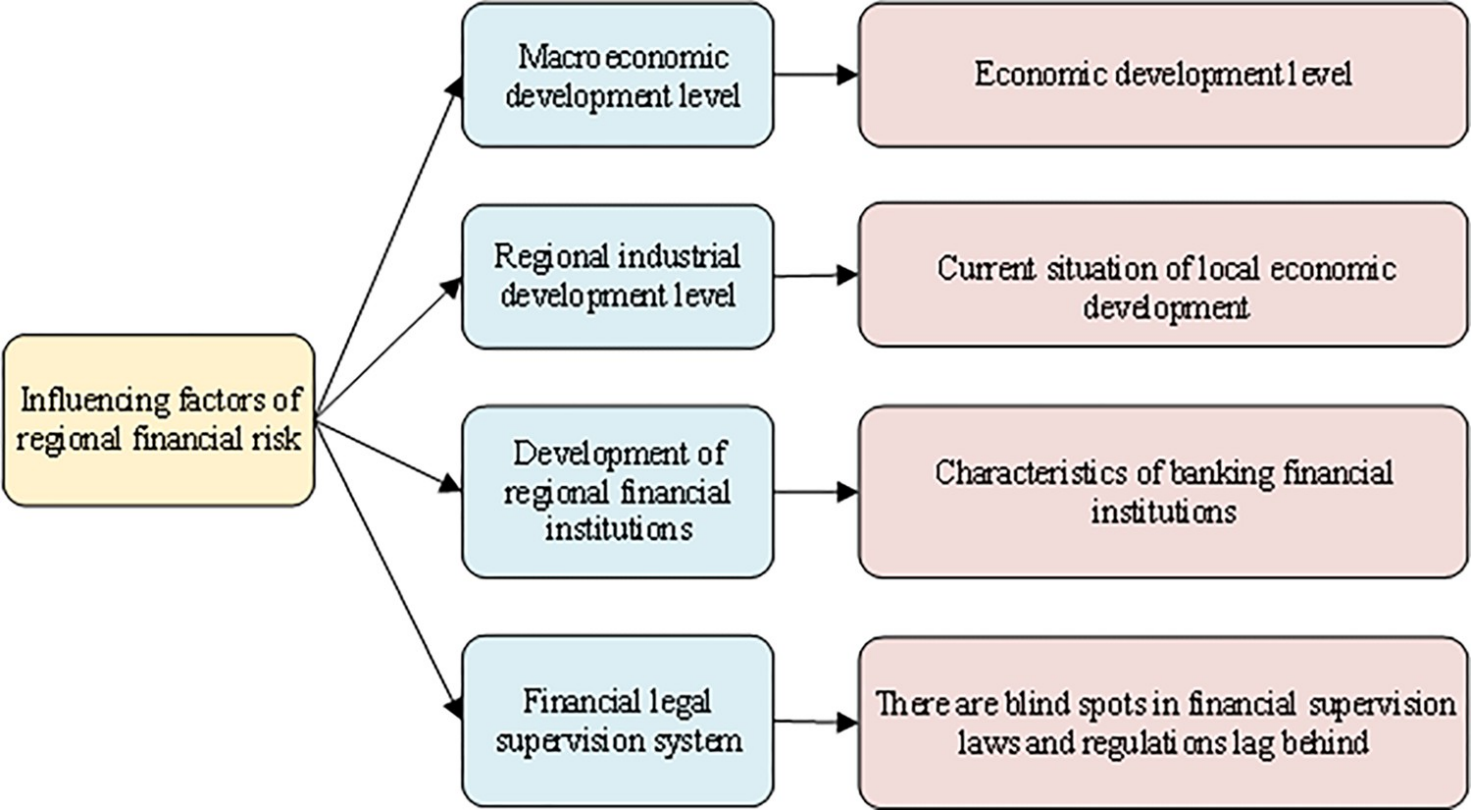
Influencing factors of RFRs.

Regional financial risks mainly consider the impact of four aspects: the level of macroeconomic development, the level of regional industrial development, the development of regional financial institutions, and the financial legal supervision system [17]. The details are shown in Fig 1.

As shown in Fig 1, from the perspective of the macroeconomic level, economic risk is an essential factor in financial risks. RFRs is closely related to the market macroeconomic factors of stocks, bonds, currencies, and real estate. The economic slowdown, the industrial structural transformation, the real estate market downturn, and investors’ lack of confidence will all affect regional finance’s development. Local economic development indirectly affects regional financial stability. Regional financial institutions are mainly banks. If the non-performing loan ratio of the banking industry increases, it will induce a run on the banking industry, which will easily induce RFRs. Loopholes in the financial and legal supervision system, the applicable fuzzy boundary of legal norms, and the lack of governance and authority of local regulations are also important factors causing RFRs.

#### 2. Financial risk incentive theory

**Fig 2:**
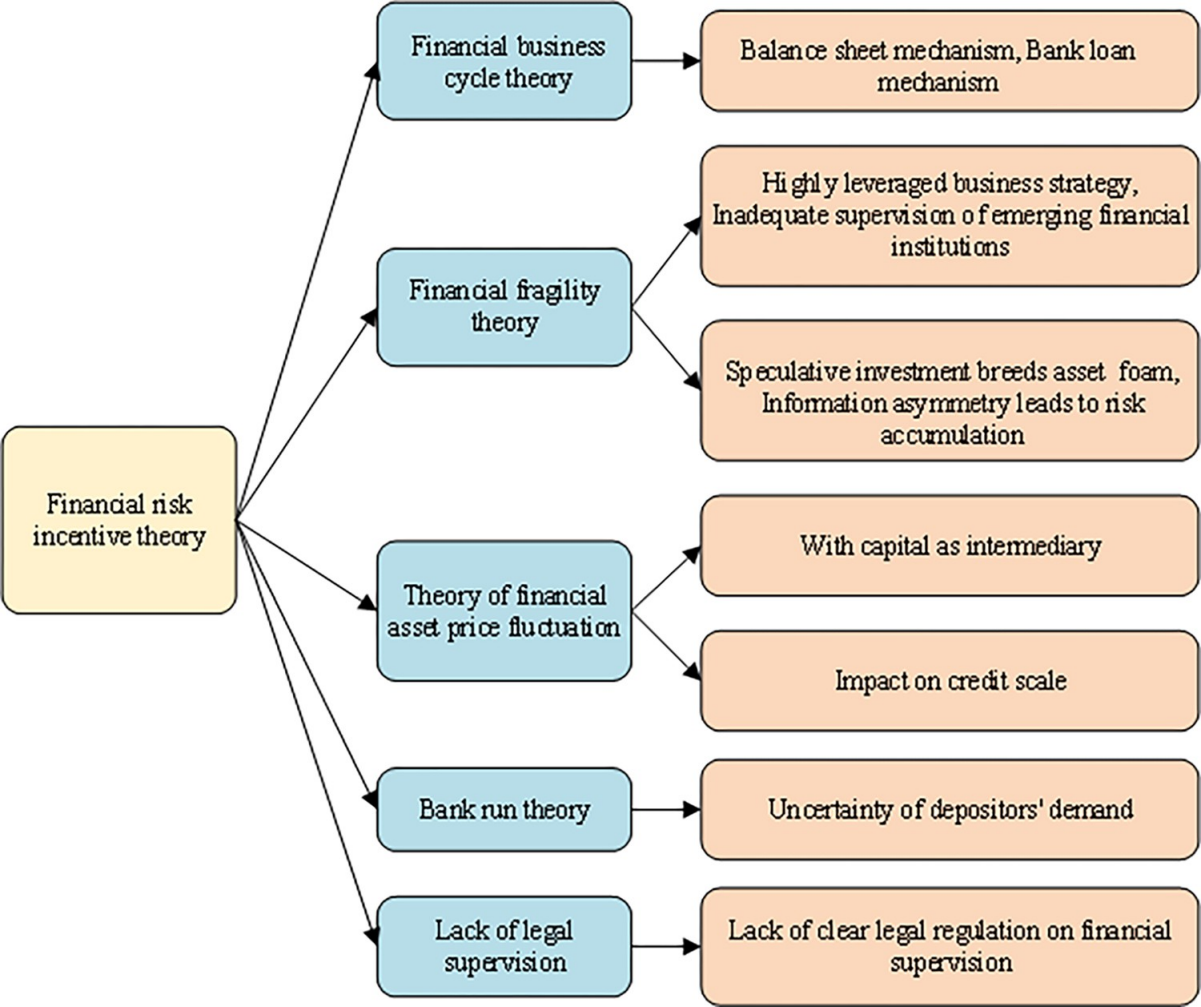
The financial risk incentive theory.

The theory of financial risk incentives includes five aspects: financial business cycle theory, financial vulnerability theory, financial asset price fluctuation theory, bank run theory, and lack of legal supervision [18]. Fig 2 shows the details.

As shown in Fig 2, the financial business cycle refers to the periodic changes in financial and economic operation under the joint action of internal and external factors. From the perspective of financial development, this work studies the dynamic change mechanism in different stages of the financial cycle. The balance sheet and bank loan mechanism are the primary factors that increase financial risks. The theory of financial fragility refers to the accumulation of internal risks formed by its high debt business model. The risks mainly come from the highly leveraged business strategy, the lack of legal and regulatory constraints of emerging financial institutions, the growth of asset foam caused by speculative investment, and the accelerated accumulation of risks caused by information asymmetry. The fluctuation law of financial asset prices also differs significantly from the trend of macroeconomic operation. The banking industry’s assets mainly come from the deposits of depositors, which makes the liquidity of bank assets worse than that of liabilities. When banks realize asset appreciation in the form of short deposits and long loans, the uncertainty of depositors’ demand is the main factor causing bank runs. The lack of legal supervision is the lack of clear legal regulation of financial supervision, imperfect financial supervision regulations, and lack of legal texts for risk prevention and control.

#### 3. The transmission channel of RFRs

**Fig 3:**
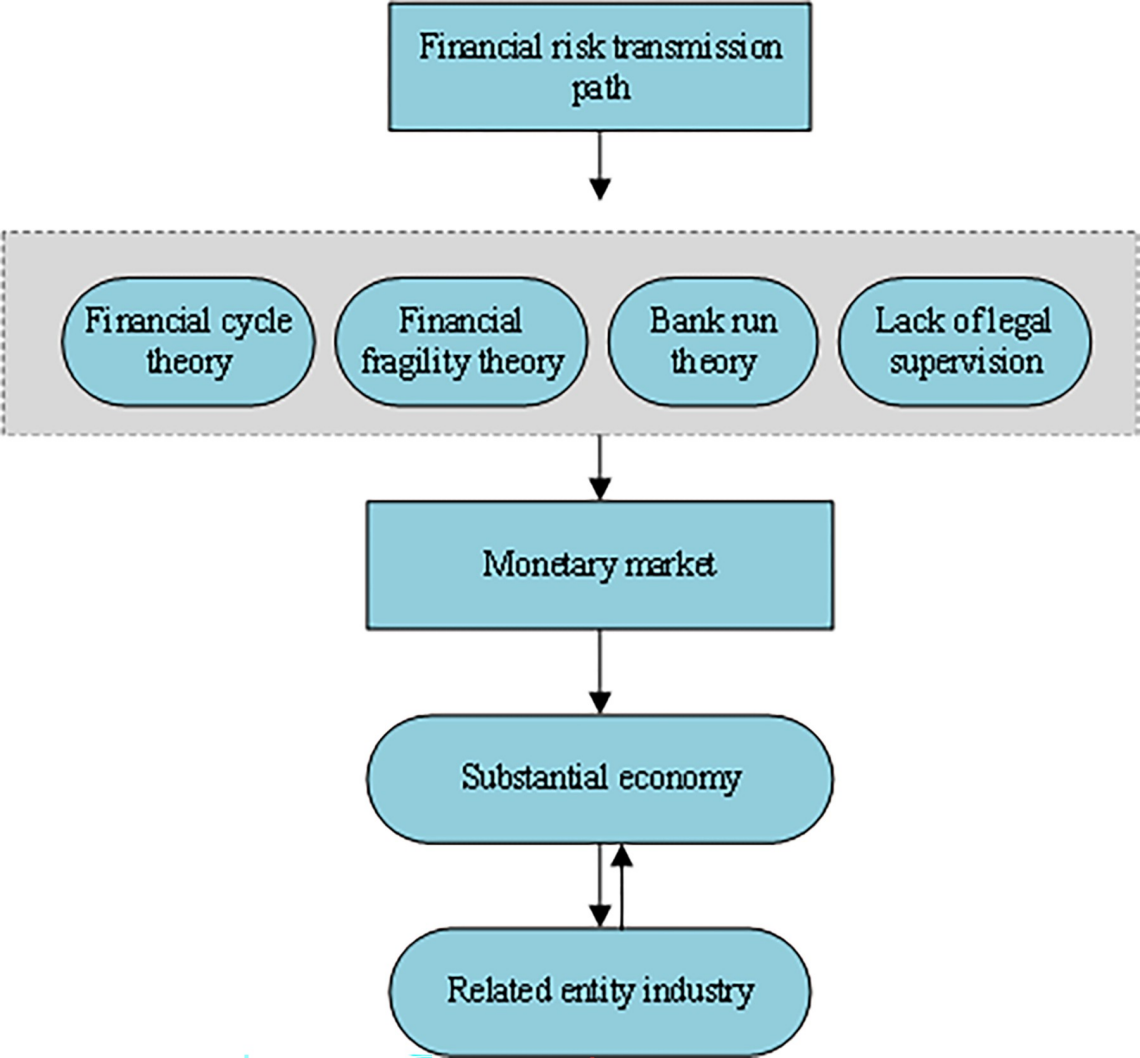
RFRs transmission channels.

The financial risk transmission mechanism mainly includes the transmission of trade, financial channels, and two-way transmission between the real and financial industries. The main transmission path is the two-way transmission between the real and financial industries [19]. The specific transmission path is demonstrated in Fig 3.

According to Fig 3, trade channel transmission mainly refers to the risk transmission between two regions with trade exchanges. Financial channel transmission refers to the outbound regional capital transfer induced by financial risks through capital flow, bank lending, and financial product investments. Meanwhile, unclear definitions of FM laws and regulations and the division of supervision power unregulated by legal norms and guidelines make the entire FM the most important channel for risk transmission. Essentially, the two-way transmission mechanism between the real economy and the financial industry is the two-way risk transmission of financial risks through the real economy and the FM.

### RFRs-oriented EW system

#### 1. DL model theory

**Fig 4:**
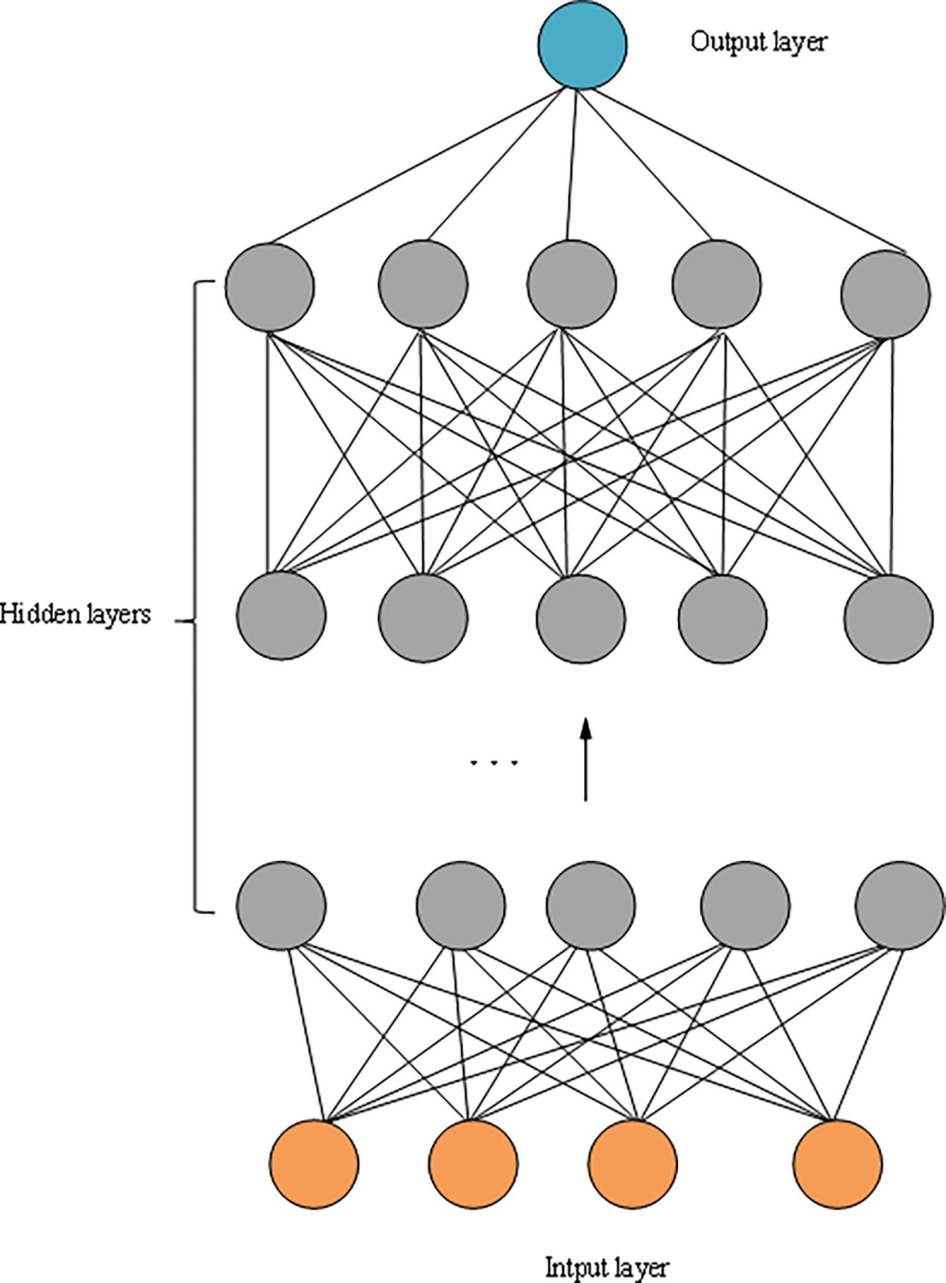
DNN model.

Deep Neural Network (DNN) is an effective machine learning algorithm for DL. It learns the inherent laws and representation levels of sample data. It can automatically learn data features and complete tasks such as classification and regression [20]. Its ultimate goal is to enable the machine to have the same analysis and autonomous learning ability as people and recognize characters, images, and other data [21]. DL extracts features layer by layer by mining the underlying feature distribution of the data, with multiple hidden layers. The hidden layer connects the input and output layers. The structure of the DNN model is unfolded in Fig 4.

As explained in Fig 4, unsupervised learning from the input layer to the output layer is used in the DNN model. DNN starts from the input layer and trains layer by layer to the top layer. The parameters of each layer are trained layer by layer without calibration data. This training method can be regarded as an unsupervised training process [22]. The idea of DL feature extraction is applied to the construction of financial risk EW model. The DL data is used to mine features, analyze regional financial risk indicators, and mine financial risk and influencing factor indicators.

#### 2. RFRs-oriented EW model

The traditional financial risk EW model includes the Frequency Ratio (FR) model. FR is based on the influencing factors of currency risk. The FR model expressed in Eq (1):

$$\begin{array}{c} P(Y=1)=F(x,q)\\ P(Y=0)=1-F(x,q) \end{array}$$ (1)

In Eq (1), *Y* is the financial risk variable. *Y* = 1 indicates the outbreak of financial risk. *x* represents the influencing factor of financial risk. *q* denotes the parameter vector of *x*. The joint probability of induced variables is used to measure the outbreak probability of financial risk. Suppose N countries are measured, and the sample period is 1,2,⋯,*T*. In that case, *p*{*i*, *t*} is estimated by Eq (2) [23]:

$$p\{i,t\}=\left\{ \begin{array}{l} 1,\;When\;risk\;occurs\;in\;country\;i\;at\;moment\;t\\ 0,\;When\;no\;risks\;occur\;in\;country\;i\;at\;moment\;t \end{array}\right.$$ (2)

The comprehensive result of country *i* in period *t* is *x*{*i*, *t*}.

MS-VAR model can well analyze the structural changes between variables and predict the future data change rules based on summarizing the historical data change rules. The specific model is as follows:

The vector autoregressive model composed of the *k*-dimensional time series *y*_*t*_ = (*y*_1*t*_,⋯,*yk*_*t*_)′ is expressed by Eq (3):

$$y_{t}=v+A_{1}y_{t-1}+\cdots +A_{p}y_{p-1}+\mu_{t}$$ (3)

In Eq (3), *t* = 1,2,⋯,*T*, *μ*_*t*_~*IID*(0,Σ), and *y*_0_,⋯,*y*_*t*−*p*_ are the determined variable. Suppose the error variable conforms to the normal distribution, or *μ*_*t*_~*IID*(0,Σ). In that case, Eq (3) can be expressed as the intercept term of *VAR*(*p*) model. Its expression is given in Eq (4) [24]:

$$y_{t-1}-\mu =A_{1}(y_{t-1}-\mu_{t})+\cdots +A_{p}(y_{t-p}-\mu )+\mu_{t}$$ (4)

In Eq (4), *μ* is the *k*×1 mean form of *y*_*t*_. The calculation of *μ* reads:

$$\mu ={\left(I_{k}-\sum_{j=1}^{p}\;A_{J}\right)}^{-1}v$$ (5)

When the time series is affected by the change in the regime system, it is assumed that the regime switch variable *S*_*t*_∈{1,⋯,*M*} is a Markov chain in a discrete state. The conversion probability is counted by Eq (6):

$$\begin{array}{c} P_{ij}=Pr(S_{t-1}=j|S_{t}=i)\\ \sum_{j=1}^{M}\;P_{ij}=1\\ \forall i,j\in \{1,\cdots ,M\} \end{array}$$ (6)

When the order of the MS-VAR model is *P*, the specific performance of the Regime Switch Model (SRM) reads:

$$y_{t}-\mu (S_{t})=A_{1}(S_{t})(y_{t-1}-\mu (S_{t-1}))+\cdots +A_{p}(S_{t})(y_{t-p}-\mu (S_{t-p}))\mu_{t}$$ (7)

In Eq (7), *μ*(*S*_*t*_), *A*_1_(*S*_*t*_), and *A*_*p*_(*S*_*t*_) are parameters of *μ*. *A*_1_⋯,*A*_*p*_ is applicable to the parameter function of regime *S*_*t*_. *μ*(*S*_*t*_) is calculated by Eq (8):

$$\mu (S_{t})=\left\{ \begin{array}{c} \mu_{1}S_{t}=1\\ \vdots \\ \mu_{M}S_{t}=M \end{array}\right.$$ (8)

The RSM is added to the intercept term to achieve a reasonable state of regime switch and mean smooth switch [25]. The results are reflected in Eq (9):

$$y_{t}=v(S_{t})+A_{1}(S_{t})y_{t-1}+\cdots +A_{p}(S_{t})y_{t-p}+\mu_{t}$$ (9)

### RFRs-oriented EW index and its Composite Index (CI) construction

#### 1. RFRs-oriented EW index

**Fig 5:**
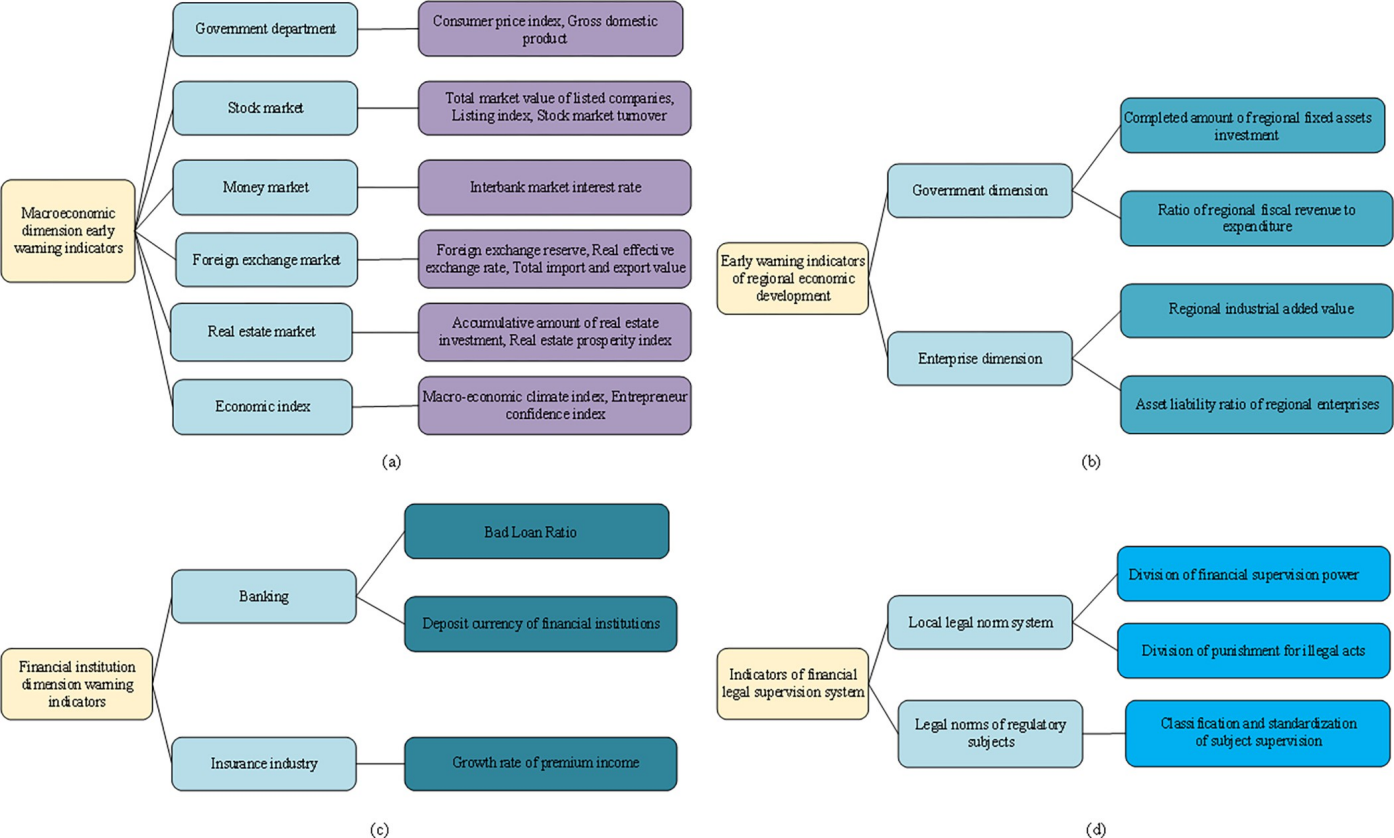
RFRs-oriented EW indexes. (a) Macroeconomic operation risk EW index; (b) Regional economic risk EW index; (c) Regional financial institution risk EW index; (d) Financial legal supervision system risk EW index.

This section takes Shanghai as an example to construct the RFRs-oriented EW indexes from four aspects: macroeconomic operation risk, regional economic risk, regional financial institution risk, and financial and legal supervision system risk [26]. Macroeconomic operation index data are selected from government departments, stock market, bond market, and foreign exchange market [27]. Regional economic risk index data take the dimensions of government departments and enterprises as secondary indexes. The regional financial institution index considers the banking and insurance industries as secondary indexes. By comparison, the financial and legal supervision index takes the local legal norm system and the legal norm of the supervision subject as the secondary indexes. The index selection is specified in Fig 5.

Macroeconomic fluctuation is a cyclical change from depression to recovery and then to climax. In terms of the leading index, economic indexes represented by macroeconomic prosperity and entrepreneur confidence index are selected to improve the RFRs-oriented EIS further. Regional economic risk is mainly reflected in the transfer of local debt risk to the risk of the financial system and the risk transfer from the real industry to the financial system. The strength of risk management and control of financial institutions directly affects the stability of the FM, with banking and insurance as secondary indexes. The local legal norm system is reflected in two aspects: the division of financial supervision power and the division of punishment for illegal acts. The legal norms of the supervision subject are embodied in the classification and standardization of the supervision subject.

#### 2. RFRs CI

**Fig 6:**
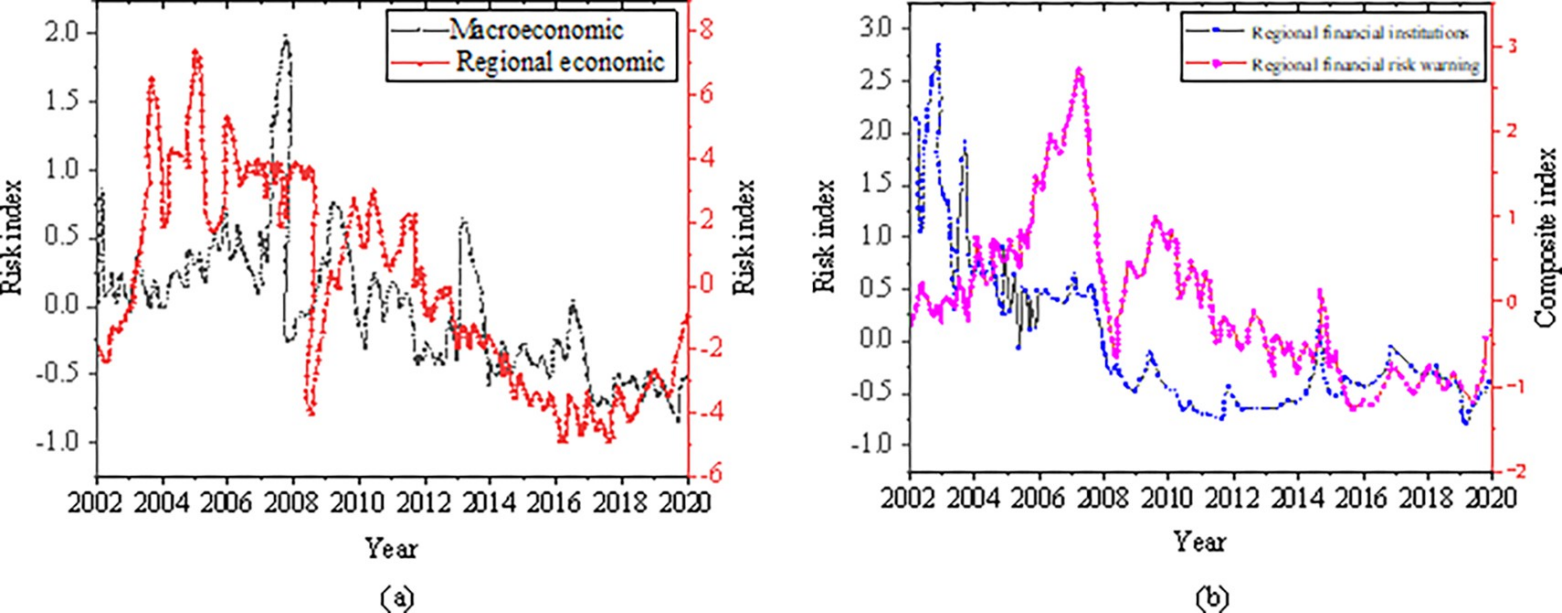
RFRs CI. (a) Economic risk index; (b) RFRs index.

In view of the characteristics of China’s financial system and the regional characteristics of financial risks, taking Shanghai as an example, this paper analyzes the macroeconomic and regional economy, regional financial institutions and regional financial risk index from 2002 to 2020. The data are derived from Wind, eastmoney.com and cnfin.com and other relevant economic and financial websites. Against the characteristics of China’s financial system and the regional characteristics of RFRs, the CI method is used to construct the RFRs-oriented EIS. It is used as the basic variable of the financial risk EW model [28]. The CI method can be converted according to variable changes and combined with various risk identification and EW models. Fig 6 lists the details.

Fig 6(A) is the macroeconomic and regional economic risk index from 2002 to 2020, and Fig 6(B) is the regional financial institution and regional financial risk index from 2002 to 2020. The economic risk index and the regional financial risk index are variable. With the strengthening of government supervision, the risk index has decreased.

### DL-based MS-VAR model for RFRs EW

Combined with the DL model, the regional financial risk EW indicators are analyzed, and on this basis, the regional financial risk EW MS-VAR model is constructed. In order to strengthen the EW ability of the MS-VAR model, EW inspections are carried out on risks of different dimensions, that is, risk state transition identification is carried out, and transition probability identification is carried out for three different risk levels. It includes low risk level, medium risk level, and high risk level [29]. The process is as follows:

$$r_{t}=\mu_{t}+\varepsilon_{t}{,\varepsilon }_{t}∼N(0,\sigma_{t}^{2})$$ (10)

$$\mu_{t}=\sum_{i=1}^{3}\;\mu_{i}S_{it}\sigma_{t}^{2}=\sum_{i=1}^{3}\;\sigma_{i}^{2}S_{it}$$ (11)

$$p_{ij}=Pr(S_{t}=j|S_{t-1}=i,S_{t-2}=k,S_{t-3}=l,\cdots \cdots )=Pr(S_{t-1}=i)$$ (12)

$$S_{it}=\left\{ \begin{array}{c} 1,\;S_{t}=i\\ 0,\;others \end{array}\right.$$ (13)

In Eqs (10)—(13), *S*_*t*_ is the state variable. *S*_*t*_ = 1,2,3 respectively represent a low-level financial risk, medium level financial risk, and high-level financial risk. {*S*_*t*_} denotes a first-order Markov chain. The state variable *S*_*t*−1_ at the previous moment determines *S*_*t*_ at moment *t*. *p*_*ij*_ stands for state conversion probability.

The conversion probability matrix *P* of *S*_*t*_, is obtained by Eq (14):

$$P=\left[\begin{array}{c} p_{11}\;p_{12}\;p_{13}\\ p_{21}\;p_{22}\;p_{23}\\ p_{31}\;p_{32}\;p_{33} \end{array}\right]$$ (14)

In Eq (14), *p*_*ij*_∈[0,1], and $\sum_{i=1}^{3}\;p_{ij}=1,$ *i* = 1,2,3.

*I*_*t*−1_ indicates the information set of *r*_*t*_ corresponding to moment *t*−1. Then, the joint density function is expressed by Eq (15):

$$f(r_{t}|I_{t-1})=\sum_{S_{t-1}}^{3}\;\sum_{S_{t-1}=1}^{3}\;f(r_{t}|S_{t},S_{t-1},I_{t-1})\cdot Pr(S_{t},S_{t-1}|I_{t-1})$$ (15)

Suppose *r*_*t*_ is known. In that case, the change of joint distribution probability is calculated by Eq (16):

$$\begin{array}{l} Pr(S_{t}=j,S_{t-1}|I_{t})\\ \;=Pr(S_{t}=j,S_{t-1}=i|I_{t-1},r_{t})\\ \;=\frac{\mathrm{Pr}\left(S_{t}=j,S_{t-1}=i,r_{t}|I_{t-1}\right)}{f(r_{t}|I_{t-1})}\\ \;=\frac{f(r_{t}|S_{t}=j,S_{t-1}=i,I_{t-1})\cdot Pr(S_{t}=j,S_{t-1}=i|I_{t-1})}{\sum_{S_{t-1}}^{3}\;\sum_{S_{t-1}=1}^{3}\;f(r_{t}|S_{t}=j,S_{t-1}=i,I_{t-1})\cdot Pr(S_{t}=j,S_{t-1}=i|I_{t-1})} \end{array}$$ (16)

The filter probability Pr(*S*_*t*_ = *j*|*I*_*t*_) is converted, and the result is given in Eq (17):

$$Pr(S_{t}=j|I_{t})=\sum_{i=1}^{3}\;Pr(S_{t}=j,S_{t-1}|I_{t})$$ (17)

Based on the filter alternation effect, the smoothing probability Pr(*S*_*t*_ = *j*|*I*_*t*_) is calculated. The smaller the smoothing probability estimation is, the smaller the probability that the moment *t* is at the *i*th volatility level [30].

## Empirical research and analysis on risk EW

### Economic pressure CI

**Fig 7:**
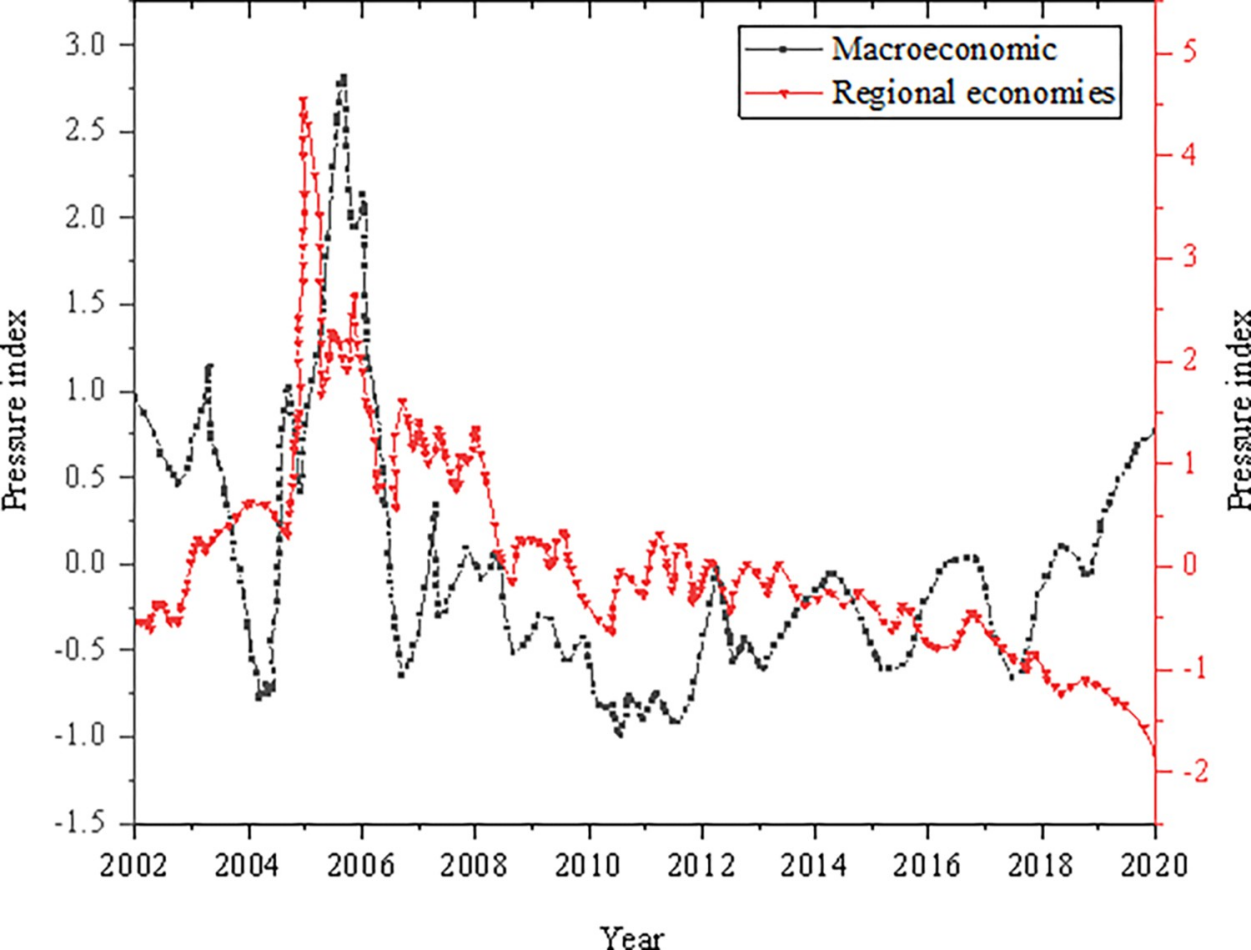
Economic pressure CI.

The results are shown in Fig 7 from the macro-economy and regional economy perspectives.

As explained in Fig 7, the macroeconomic pressure index has a large fluctuation trend in the time series. The macro-economy is subject to external shocks, and the pressure posed by potential risks continues to accumulate. When the impact utility expands rapidly, a pressure risk erupts. The overall fluctuation of the regional economic pressure index is small. It fluctuates up and down at 0 in most periods, and the larger fluctuation range appears around 2004. In particular, the fluctuation trend of the regional economic pressure index is different from the macroeconomic dimension. This is mainly because the impact of the global financial crisis on regional economic development is significantly less than the macroeconomic fluctuation.

### Regional financial pressure CI

**Fig 8:**
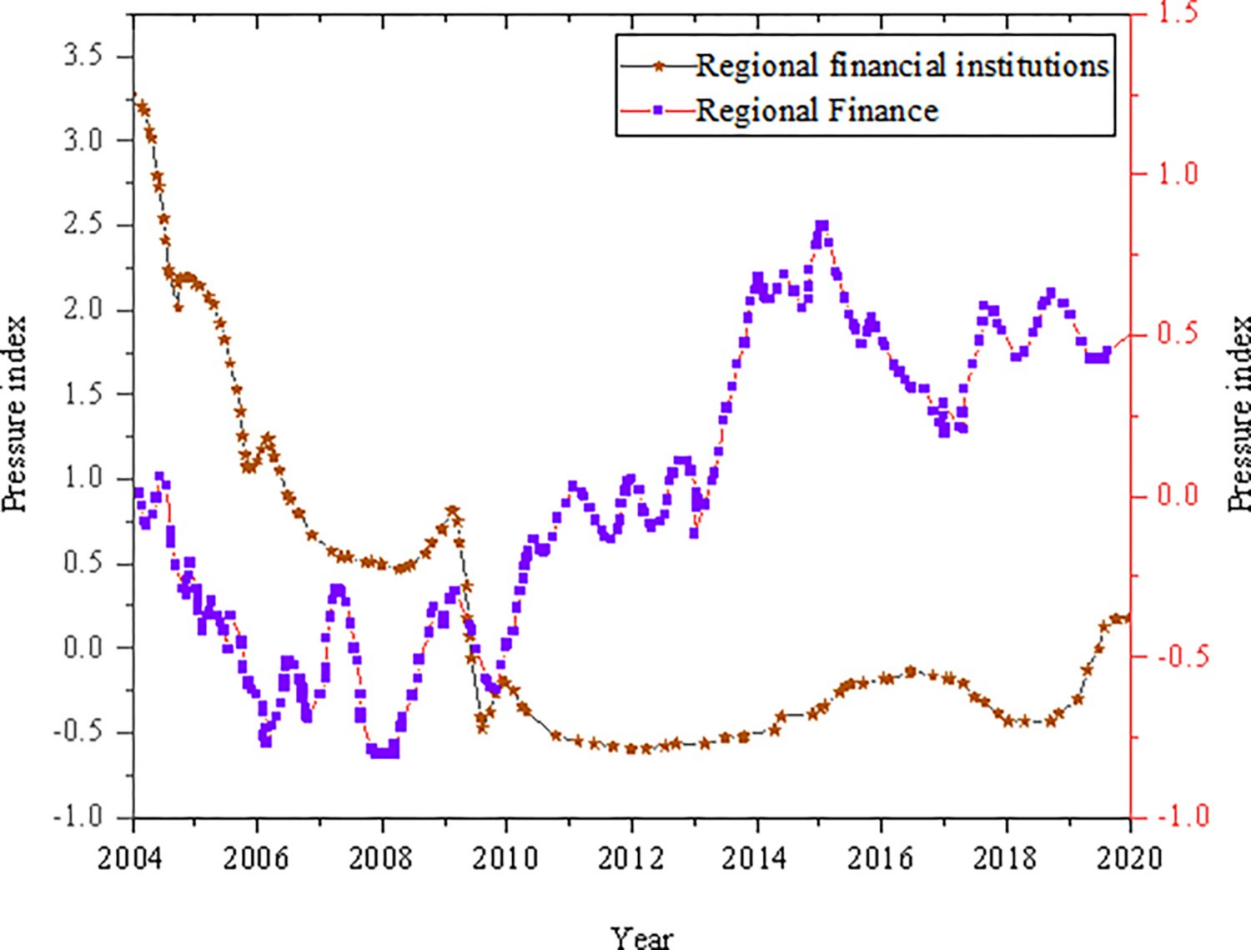
Financial pressure CI.

Fig 8 analyzes the regional financial pressure CI from the two aspects of regional financial institutions and regional banking.

As shown in Fig 8, the fluctuation range of the dimensional pressure index of regional financial institutions is significantly higher than that of the regional banking dimension. From 2004 to 2010, the non-performing loan ratio of the banking industry was always at a high level. After the financial crisis, the banking risks were released to a certain extent, and the legal supervision of local governments was strengthened. At the same time, the supervision of non-performing enterprises and residents’ loans was strengthened, which greatly reduced the non-performing loan ratio of the regional banking industry. From 2011 to 2018, the non-performing loan ratio began to decline, and the pressure index of financial institutions entered a gentle state. The overall fluctuation range of the regional financial pressure CI is small, floating up and down the zero value. The financial pressure CI has changed more dramatically during 2004–2006, 2007, 2009, and 2016.

### MS-VAR EW inspection

**Fig 9:**
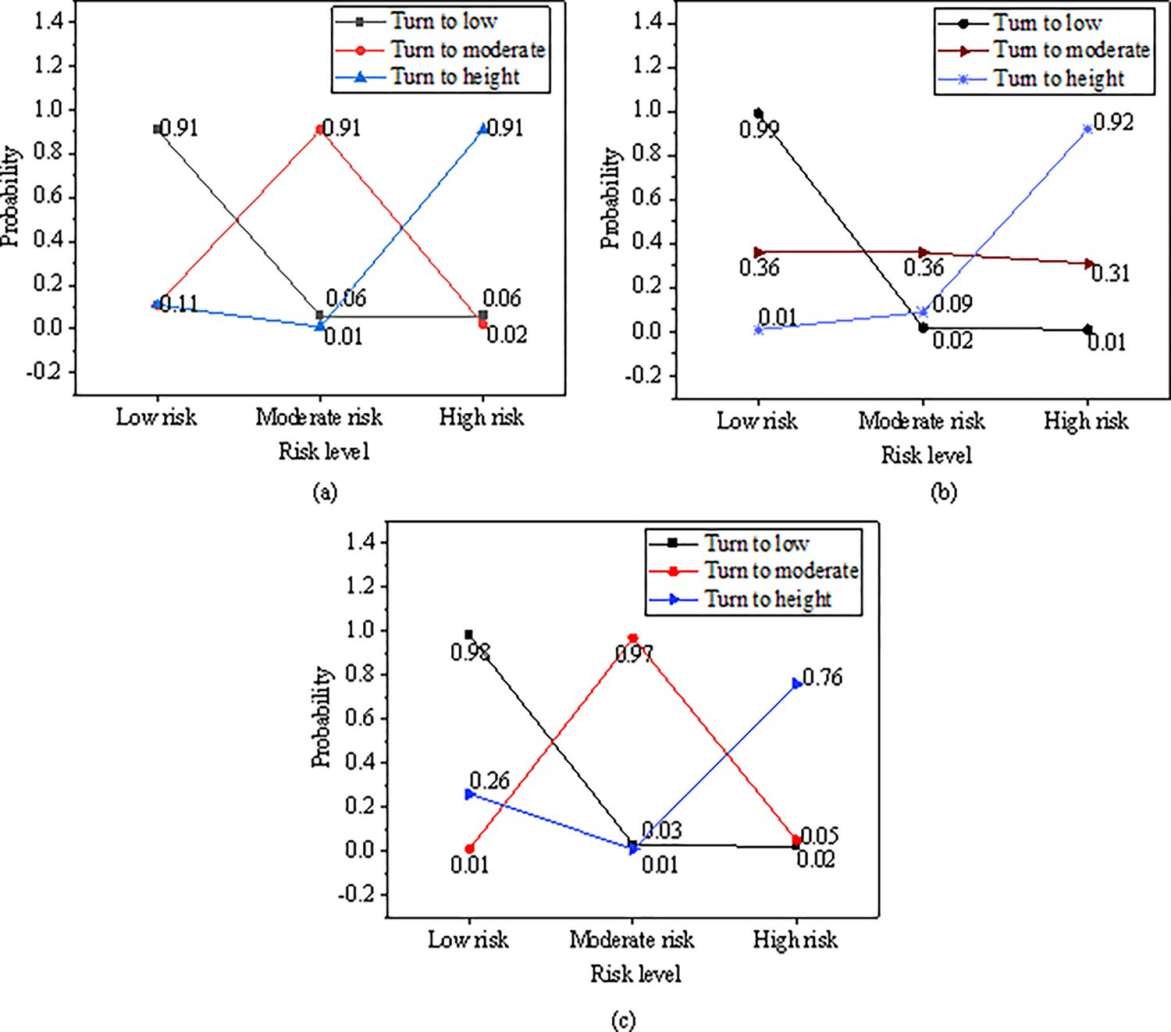
EW test of financial risks in different dimensions. (a) Conversion probability of macroeconomic risk level; (b) Conversion probability of regional economic risk level; (c) Conversion probability of regional financial structure dimension.

EW tests are performed on risks of different dimensions. Risk state conversion identification is mainly based on three levels: high risk, medium risk, and low risk, as revealed in Fig 9.

As revealed in Fig 9, in terms of the macroeconomic dimension, the probability of maintaining the same state of transition among the three regional systems of low, medium and high risk is all 0.91. This indicates that the stability of the transition between regional systems is relatively strong, and the probability of maintaining the original risk level is high. It is easier to convert regional economic risk from low risk to moderate risk, but it is more difficult to convert from high risk to moderate risk. From the perspective of regional financial institutions, the probability of maintaining low risk, medium risk, and high risk is 0.98, 0.97, and 0.76, respectively, and the stability of low risk and medium risk is stronger than that of high risk.

### State conversion identification of RFRs CI

**Fig 10:**
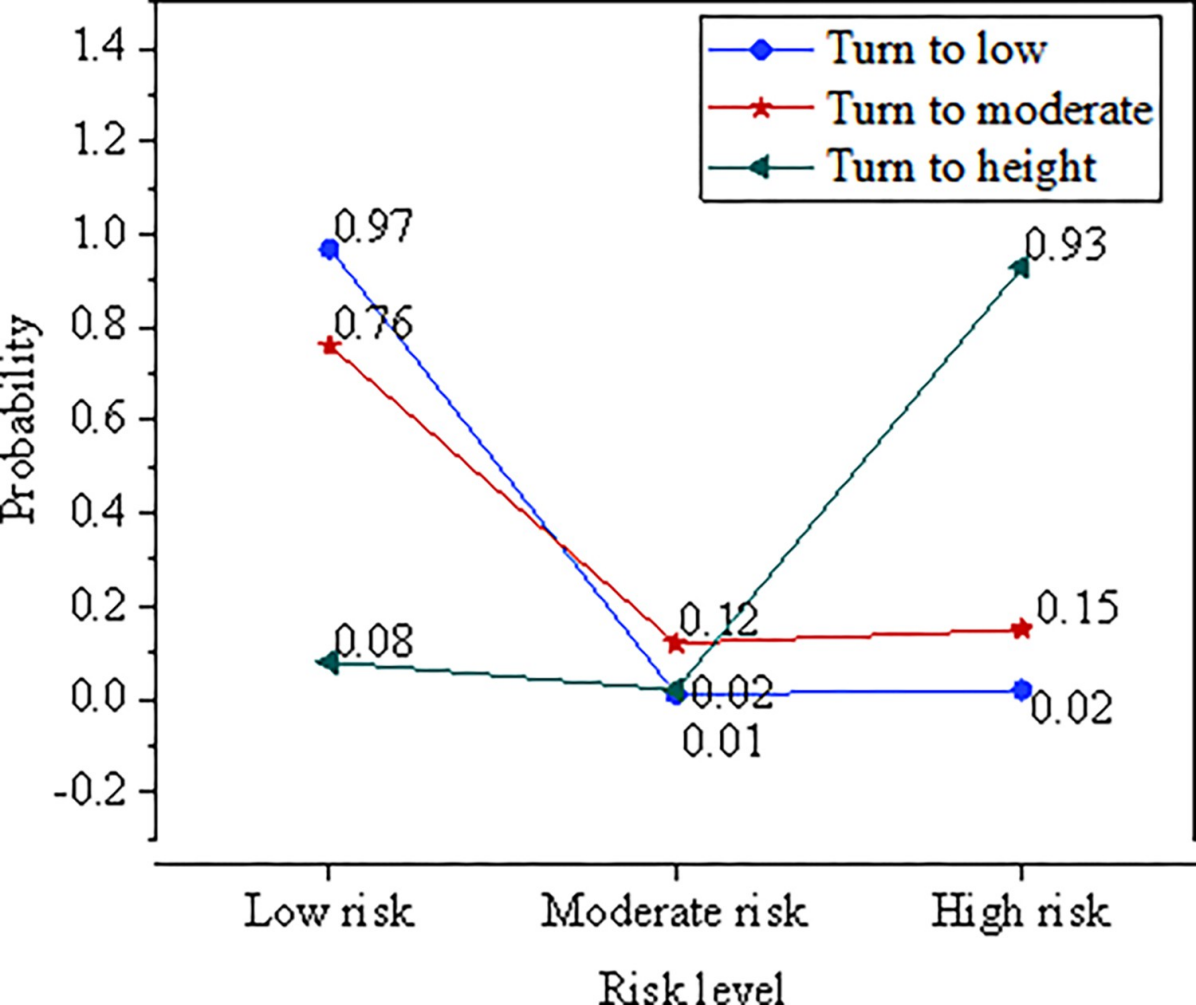
Conversion probability between different risk Cis.

The state conversion identification of RFRs CI is described in Fig 10.

As described in Fig 10, the probability of maintaining low risk is 0.97, and the probability of maintaining high risk is 0.93. The stability of the two-zone system is strong, but the probability of maintaining moderate risk is less than 0.3, indicating that the moderate risk fluctuates greatly. The dimension mainly analyzes the state transition probability of low risk and high risk. The probability of high risk and low risk level conversion is high.

### RFRs prediction

#### 1. Prediction test of regional financial pressure index

**Fig 11:**
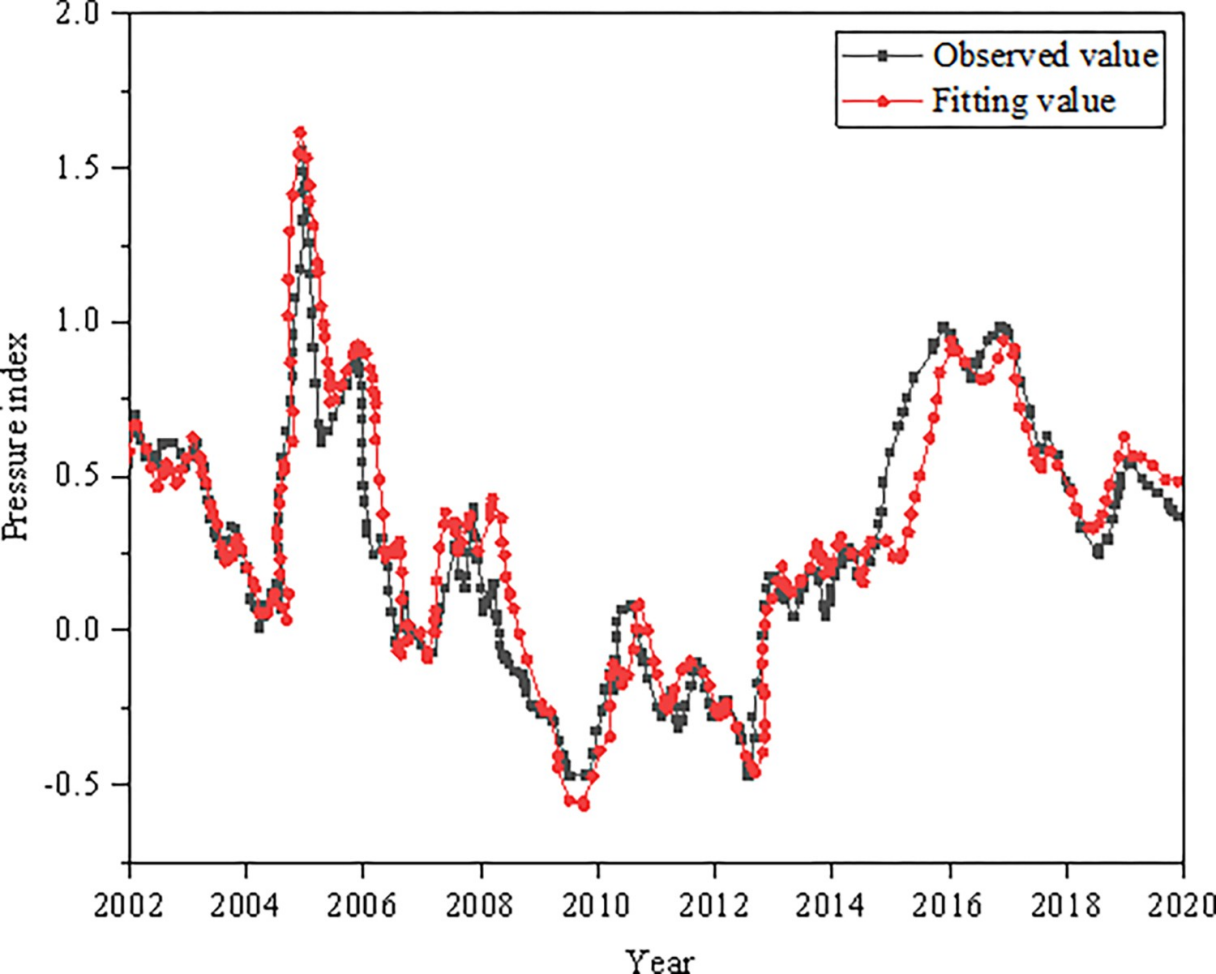
Fitting results of regional financial pressure index.

Based on Shanghai, the fitting trend of the regional financial pressure index from 2002 to 2020 is analyzed in Fig 11.

As illustrated in Fig 11, the observed value of the fitted regional financial risk EW comprehensive pressure index has a high degree of coincidence with the fitted value, and the observed value and the fitted value curve have a good agreement as a whole, indicating that the DL regional financial risk EW MS-VAR model has strong predictive ability and high credibility of the predicted data.

#### 2. Prediction test for RFRs

**Fig 12:**
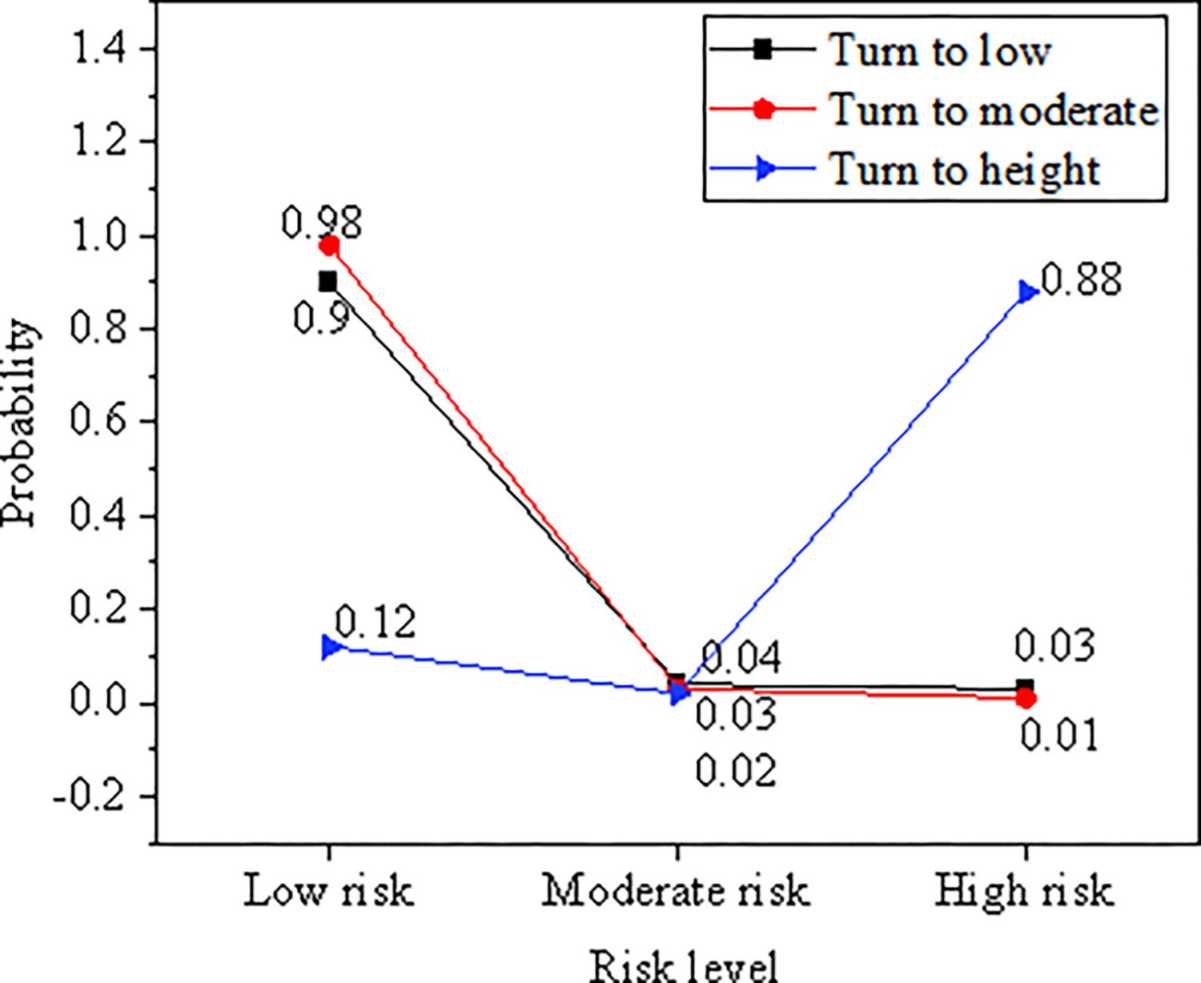
Conversion probability of RFRs CI.

The proposed RFRs-oriented EW model is used to estimate the three-regime conversion probability of the RFRs CI. The results are plotted in Fig 12.

According to Fig 12, the probability of low risk maintaining low is 0.90, and the probability of high risk maintaining high is 0.88. The stability of the two-regime system is strong, but the probability of medium risk conversion is relatively low. Thus, the fluctuation of medium risk is large. Therefore, the conversion probability of risk CI mainly analyzes low risk and high risk. The probability of conversion from low risk to medium risk is high. The regional financial risk EW MS-VAR model of DL can better analyze the conversion probability of regional financial risk EW index.

## Discussion

Based on the DL model and MS-VAR model, this paper constructs the regional financial risk EW model from three aspects: macroeconomic operation EW index, regional economic risk EW index, and regional financial institution risk EW index. The model is empirically studied and analyzed. The results show that the macroeconomic pressure index fluctuates greatly in time series, while the regional economic pressure index fluctuates slightly in general, and fluctuates around 0 in most periods. From 2011 to 2018, the non-performing loan ratio began to decline, and the overall regional financial comprehensive stress index fluctuated slightly, fluctuating around 0. EW MS-VAR model of DL regional financial risk has strong risk prediction ability. The model can well analyze the conversion probability of regional financial risk EW indicators and has good risk EW ability. In this research direction, literature [23] gives EW of regional financial risks based on macroeconomic indicators, analyzes the changing trend of macroeconomic indicators by constructing multiple regression models, predicts possible economic crises and financial risks, and provides EW information for the government and financial institutions. Literature [24] constructs a technical analysis model through stock index, bond price and exchange rate, analyzes the trend of market indicators, predicts possible fluctuations and risks in the market, and provides reference for investors and financial institutions. The method in literature [25] has high accuracy. By constructing multi-dimensional evaluation model and supervision index system, the risk status of financial institutions is analyzed and forewarned.

Compared with these studies, the advantages of this paper lie in the following points. First, risk characteristics can be captured more accurately. DL model and MS-VAR model can capture the nonlinear relationship and state switching characteristics of data more accurately to describe the characteristics of regional financial risks more accurately and improve the prediction accuracy. Second, data processing is more flexible. The EW model of regional financial risk adopts deep learning model and MS-VAR model, which can deal with nonlinear, heterogeneous and multivariable data flexibly to better meet the forecasting needs of different data types. Then, it is more interpretable. Compared with the traditional economic model, the deep learning model and MS-VAR model are more explanatory, can present the forecast results intuitively, and better provide decision support for decision makers. Finally, the EW model of regional financial risk can be flexibly adjusted and optimized according to the changes of data and forecast demand, and has stronger adaptability.

## Conclusion

### Experimental result

In order to improve the laws and regulations of the financial system and optimize the regional financial risk EW model, this paper constructs the regional financial risk EW indicators from four aspects: macroeconomic operation EW indicators, regional economic risk EW indicators, regional financial institution risk EW indicators and financial legal supervision system. According to the DL algorithm idea, the financial risk EW indicators are analyzed, the indicator system is improved, and the MS-VAR model is constructed. Finally, the regional financial risk EW MS-VAR model based on DL is constructed. Taking Shanghai as the research object, the model is empirically researched and predicted from several aspects, such as the comprehensive index of economic pressure, the comprehensive index of regional financial pressure, the MS-VAR EW test, and the comprehensive index of regional financial risk. The results show: (1) The overall fluctuation of the regional economic pressure index is small, fluctuating around 0 in most periods, and the periods with large changes in the pressure index are mainly from 2004 to 2006, 2007, 2009 and 2016. The macroeconomic pressure index fluctuates greatly in the time series, and the impact of the global financial crisis on regional economic development is less than the impact on macroeconomic development. (2) After the financial crisis, the local government increased the supervision of non-performing enterprise and household loans, which greatly reduced the non-performing loan ratio of the regional banking industry. From 2011 to 2018, the non-performing loan ratio began to decline, and the overall fluctuation of the regional financial comprehensive stress index was small, fluctuating around 0. (3) In terms of the macroeconomic dimension, the probability of maintaining risk among low, medium and high risks is all 0.91, the stability of the transition between risk zones is relatively strong, and the probability of maintaining the original risk level is relatively high. (4) From the perspective of the regional economic dimension, it is easier to convert from low risk to moderate risk, but it is more difficult to convert from high risk to moderate risk. From the perspective of regional financial institutions, the probabilities of maintaining low risk and moderate risk are 0.98 and 0.97, respectively, which is stronger than maintaining the stability of high risk. (5) From the perspective of the state transition of the regional financial risk composite index, the maintenance probabilities of high risk and low risk levels are both 0.93 and 0.97, which are higher than the maintenance probability of medium risk. From the regional financial pressure index prediction test, the overall observation value and the fitting value curve are in good agreement, indicating that the DL regional financial risk EW MS-VAR model has strong risk prediction ability. The model can better analyze the regional financial risk EW index conversion probability.

### Future research direction

Due to the limited energy, this paper still has some limitations in the study of regional financial risk EW and prevention and control based on deep learning model. The future study of regional financial risk EW can be started from the following aspects: First, it is necessary to further strengthen cross-domain cooperation and data integration capabilities. Regional financial risk involves the financial systems of many fields and countries, and it needs interdisciplinary and cross-disciplinary cooperation, integrating various relevant data and establishing a comprehensive and systematic risk EW model. Second, it is necessary to introduce advanced artificial intelligence technology and big data analysis technology. At present, artificial intelligence technologies such as machine learning and deep learning, as well as big data analysis technologies, have been gradually applied to the financial field. In the future, regional financial risk EW can also be more accurate and efficient through these technical means. In addition, it is necessary to deepen the research on the theory and method of risk EW, develop more flexible and operable EW indicators and models, and establish a sound risk EW system. Finally, it is necessary to strengthen international cooperation and information sharing and form a global risk monitoring system. Regional financial risks cross national boundaries, so international cooperation and information sharing are needed to meet the risk challenges, and a global risk monitoring system should be established to provide more effective means for preventing and resolving regional financial risks. In a word, the research direction and means of regional financial risk EW in the future should be diversified and comprehensive, which requires cross-disciplinary and interdisciplinary cooperation, giving full play to the power of scientific and technological innovation, establishing a global risk monitoring system, and providing more reliable guarantee for financial stability and economic development.

## Supporting information

S1 Data The data sets used in Figs 6–12 are all from https://datasetsearch.research.google.com/. (ZIP)
